# Pattern and Factors Associated With the Utilization of Herbs As Medications Among Patients in a Tertiary Care Hospital in Western Saudi Arabia

**DOI:** 10.7759/cureus.19502

**Published:** 2021-11-12

**Authors:** Ahmad Alamoudi, Yazeid Alrefaey, Yazeed Asiri, Eyad Farrash, Fayssal Farahat, Syed Faisal Zaidi

**Affiliations:** 1 Medicine, King Saud Bin Abdulaziz University for Health Sciences College of Medicine, Jeddah, SAU; 2 Medicine, King Saud Bin Abdulaziz University for Health Sciences College of Medicine, Makkah, SAU; 3 Infection Prevention and Control, King Abdulaziz Medical City, Jeddah, SAU; 4 Pharmacology, School of Medicine, Batterjee Medical College for Sciences and Technology, Jeddah, SAU

**Keywords:** attitudes toward using herbs, herbs, king abdulaziz medical city, jeddah, pattern of utilization of herbs

## Abstract

Background: Herbs are used worldwide as a treatment for plenty of diseases, and some herbs are used as the origin of modern medications. Although the therapeutic benefit was reported mostly from China, over time the world seemed to realize its importance. The use of herbs as medicine is also very common in the Arab region and Saudi Arabia.

Objectives: This study was conducted to assess the pattern of use of herbs as medications in Saudi Arabia and factors associated with its use.

Methods: A convenience sample of adult patients in a tertiary care hospital in Jeddah, western Saudi Arabia was used. A questionnaire consisted of two sections, i.e., demographic characteristics and the knowledge, attitudes, and practices on the use of herbs. The data collection was conducted during December 2019.

Results: Three hundred and eighty patients were included, of which 53.3% were female. Almost half (49.7%) were college graduates. More than half of patients (55.6%) used herbs themselves, and (59%) reported members of the family used herbs for the past 12 months. Most patients (62.6%) use herbs without medical consultation. Patients used herbs following information received from family members (57.8%), social media (22.5%), doctors (8.1%), books (6.6%), or TV (5%). The most-reported herb was Anise followed by cumin and cinnamon.

Conclusion: The use of herbs is common in Saudi Arabia. Herbs are mostly used for a variety of medical conditions following family members’ experience and social media recommendations. Detailed information on the type of herbs used, doses, and effects are worth further study with a focus on variation based on different regions.

## Introduction

Herbs are used worldwide as a treatment for plenty of diseases, and some herbs are used as the origin of modern medications [1]. Many countries are characterized by the traditional use of herbs to treat certain diseases [2]. Although the therapeutic benefit was reported mostly from China, over time the world seemed to realize its importance [2].

There are several examples of medical herb use. For instance, some herbs that belong to Schizandra, Astragalus, and Ligusticum species have a long history of conventional treatment in China [2]. Ligusticum and Astragalus are considered immune-enhancing, and Schizandra has been used to treat viral chronic hepatitis [2]. Another Chinese herbal medicine (CHM), called ephedra, has been proven to be effective and is used commonly to manage respiratory problems [1]. Also, CHM is known for its use to treat breast cancer [3]. They also found that CHM can be effective in minimizing the effects and symptoms associated with breast cancer medications such as post-chemotherapy disruptions like vomiting, fatigue and, diarrhea in breast cancer patients, more often in stage IV [3]. Additionally, Chinese herbs that are used in mixtures called Chinese formulations have shown anti-proliferative features that might stop the growth and reduce the possibility of metastasis and migration of cancerous cells through the body [3,4]. One study was conducted in Iran to demonstrate the positive impact of medicinal plants in the treatment of respiratory disorders and proved that plants can be a source of biological and pharmacological products for the future [5].

The use of herbs as medicine is also very common in the Arab region since they were among the first people to introduce the use of herbs for the treatment of a variety of health conditions such as epilepsy, psychological issues, and cancer [4,6]. In Saudi Arabia, the use of herbs like black seeds and aloes is common among diabetic patients, but it is not clear whether they are of any help in such conditions [6]. One study showed that the practice of traditional medicine especially the usage of herbs, which accounted for almost 29% of the study participants, is common among adult Saudi patients with neurological disorders [7]. Another study in Riyadh documented the pattern of using herbs in cancer patients, and it was highly prevalent, despite that, not many studies were conducted about the pattern of using herbs in Saudi Arabia [8].

However, the pattern of use of herbs as medications in Saudi Arabia and factors associated with its use is still not well studied. This research was conducted to describe a pattern of utilization of herbs for the treatment of medical conditions and identify sociodemographic and cultural factors associated with its use among Saudi patients.

## Materials and methods

This study was conducted in the out-patient clinics at King Abdulaziz Medical City (KAMC) in Jeddah, western Saudi Arabia. The study was conducted in December 2019. A convenience sampling technique was implemented. The sample size was estimated as 384 patients based on an assumption of 50% use of herbal among Saudi patients and a 5% margin of error at a 95% confidence interval. The sample size was increased to 420 in order to compensate for the 20% estimated non-response.

Data were collected by using a structured self-administered questionnaire. The questions were 20 multiple-choice questions with two open questions were used to explore the use and the side-effects of the herbs. The questionnaire consisted of two sections. The first part was regarding demographic characteristics, the second part was related to knowledge on the usage of herbs, attitudes, and practices toward herbs. A pilot study was applied before the start of the study on 10 patients (they were not included in the study sample) to check for readability, comprehension, question design, and length of the questionnaire. Written consent was obtained from each participant and a summary of the research was discussed before distribution of the questionnaire. The questionnaire was distributed to patients aged 18 years and above and both gender in the waiting areas.

Statistical analysis

Data were analyzed using Statistical Package for Social Sciences (version 20.0, IBM SPSS, Armonk, NY, USA). For descriptive statistics, quantitative variables like age were presented as mean and standard deviation and qualitative variables like gender and educational level were presented as frequency, percentage. For inferential statistics, we used an independent t-test for comparing means (SD) of quantitative variables, while the chi-square test was used for the comparison of qualitative variables. The level of significance was determined at a p-value < 0.05.

## Results

This study included 380 patients (response percentage was 90.5%). Males were 46.7% and females 53.3%, and almost half of them (49.7%) were college graduates. More than half of patients (n=205, 55.6%) used herbs themselves, and 217 of patients (59%) reported that members of the family used herbs for the past 12 months. However, 69.7% of all patients prefer to go to the doctor when they feel not well. Moreover, 41.6% of all patients who use herbs prefer to use them when they are sick. On the other hand, the rest of the patients use the herbs as a routine whether daily, weekly, monthly, or annually. In addition, 62.6% of all patients use herbs alone not with prescribed medication, although, 69.1% of all patients think that using prescribed medicine is better, 48.8% of all patients believe that using herbs and prescribed medicine together is better than using herbs or medicine alone. Most patients accounted of 61.9% use herbs without knowing the scientifically proven effect, because they heard about it from their family members (57.8%), social media (22.5%), doctors (8.1%), books (6.6%), or TV (5%). Also, we asked about the preferred way of knowing about the herbs they use, and most patients prefer the internet (57.8%) while others prefer handouts, consultation, or books [see tables 1,2].

**Figure 1:**
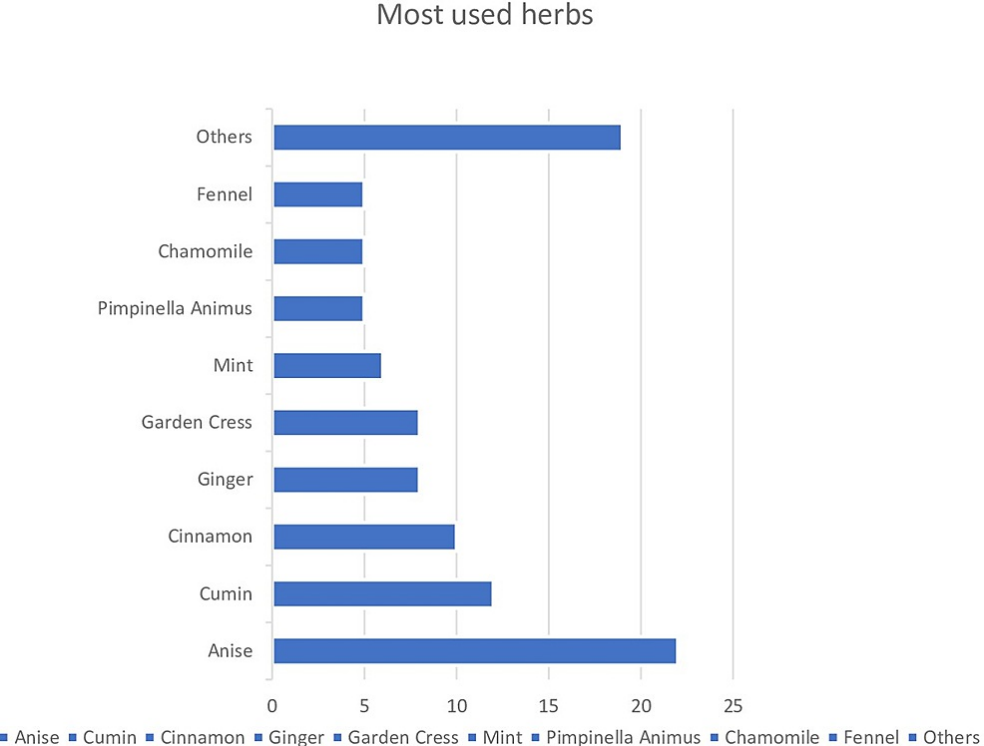
Most used herbs by participants (%) Other herbs that used in this research: Boswellia Carterii = 1, Boswellia sacra = 1, Azadirachta indica = 2, Basil = 1, Beetroot = 1, Cadaba = 1, Cardamom = 1, Carterii Boswellia = 1, Cat's claw = 2, Chia seed = 1, Cinnamomum verum = 3, Cinnamomum zeylanicum = 1, Clove = 2, Commiphora myrrha = 1, Common sage = 2, Coriander = 2, Crataegus = 2, Cuminum cyminum = 5, Curcuma longa = 1, Drumstick tree = 2, Epimedium = 1, Eucalyptus = 1, Fat with herbs = 1, Fenugreek = 2, Foeniculum vulgare = 1, Garlic = 5, Green tea with lemon = 1, Ground coffee = 2, Guava leaves = 2, Honey = 2, Khella = 1, Lemon = 7, Lemongrass = 3, Lepidium sativum = 1, M. chamomilla = 1, Mnuka honey = 1, Matricaria chamomilla = 1, Mint oil = 1, Mixture of herbs = 2, Mummies = 1, Myrrh = 6, Onion syrup = 2, Parsley = 3, Peganum = 2, Psidium guajava = 3, Roselle = 3, Rosemary = 1, Safflower = 1, Salvia miltiorrhize = 1, Salvia officinalis = 4, Salvia Rosmarinus = 1, Saussurea costus = 7, Senna = 5, Spinach = 1, Thyme = 4, Trigonella foenumgraecum = 3, Turmeric = 4, Zahra Mekky = 1, Zingiber officinale = 1, Ziziphus spina-christi = 1

**Figure 2:**
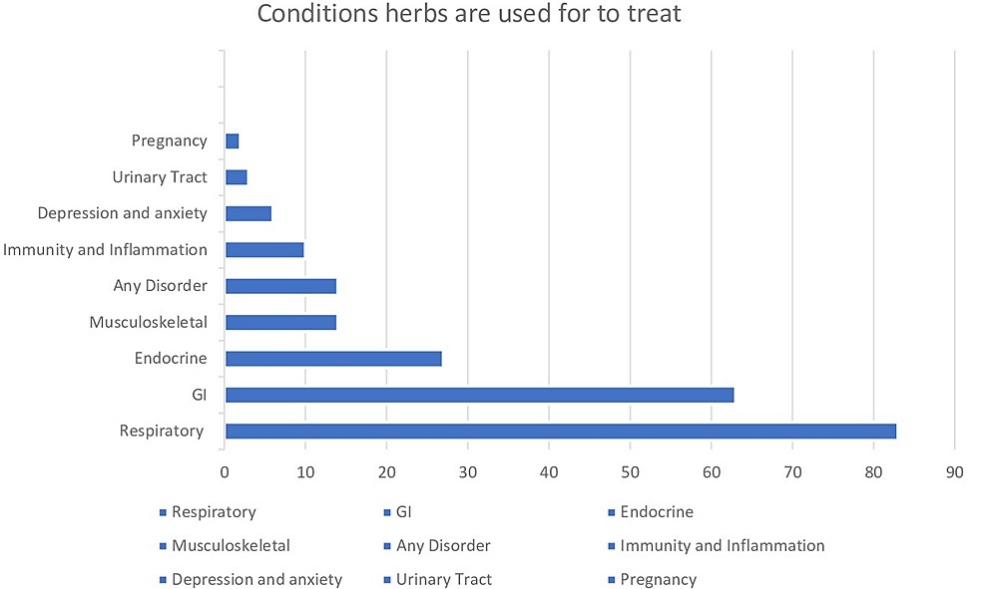
Conditions when herbs are used in treatment and number of cases.

The most-reported herb was anise followed by cumin and cinnamon. These herbs were used to treat many symptoms of respiratory symptoms such as coughing and sore throat, and gastrointestinal symptoms such as abdominal gas and nausea. Also, they used herbs for improving immunity, menstrual cycle, and bone fractures [see figures 1,2].

**Table 1:** Demographic characteristics of the study participants

Characteristics	Population in (%) out of 380 participants	P-value <0.05 is significant
Age		<0.012
18-22	57 (15)	
23-30	72 (19)	
31-40	87 (23)	
40-55	122 (31.9)	
>55	42 (11.1)	
Gender		<0.000
Male	177 (46.7)	
Female	203 (53.3)	
Education Level		<0.001
Middle school	80 (21.1)	
High school	111 (29.2)	
College or above	189 (49.7)	
Last visit to a health professional		<0.063
Less than 6 months ago	264 (69.4)	
More than six months, less than a year	44 (11.6)	
More than a year ago	72 (19)	
Self-perceived health status		<0.087
Excellent, very good. good	346 (91.1)	
Fair, poor	34 (8.9)	
During the past 12 months, did you use an herb for your own health or treatment?	YES 211 (55.6)	NO 169 (44.4)
During the past 12 months, did any of your family members use an herb for their own health or treatment?	YES 224(59)	NO 156 (41)

**Table 2:** Attitudes of herbs usage and general knowledge.

Attitudes	Population in (%) out of 380 participants		
Prefer visiting a health professional when sick	274 (72.2)		
Prefer complementary and alternative medicine when sick	106 (27.8)		
Frequency of herbs usage			
Daily	49 (13)		
Weekly	47 (12.4)		
Monthly	49 (13)		
Yearly	38 (10.1)		
Only when sick	198 (51.5)		
Use the herb with other prescribed medications	YES 123 (32.4)	NO 257 (67.6)	
Any known scientifically proven effect of the herb that was used	YES 145 (38.1)	NO 100 (26.2)	I DO NOT KNOW 135 (35.7)
Adverse reaction of any herbs	YES 40 (10.6)	NO 340 (89.4)	
Physicians acknowledge of patient herb use	YES 117 (30.7)	NO 263(69.3)	
Believe that herbs used with medications are better than alone	YES 185 (48.8)	NO 195(51.2)	
Believe that herbs are better than medications	YES 114(30.1)	NO 266 (69.9)	
How did you know about the herb you use?			
Family Members	220 (57.8)		
TV	19 (5)		
Social Media	85 (22.5)		
Books/Magazines	25 (6.6)		
Physician/Health	31 (8.1)		
the Preferred method for obtaining information about herbs:			
Internet	184 (48.5)		
Handouts	16 (4.1)		
Consultation	113 (29.8)		
Books	67 (17.5)		

## Discussion

The current results indicate that most of the studied Saudi population use herbs. The findings are similar to several studies conducted locally and internationally where the use of herbs for treatment was common [9-15]. A possible reason for people to use herbs is related to the perceived benefits, especially with long periods of use among families. Generally, people tend to believe that the use of herbs is safer than modern medicines [9,16]. This belief is not valid, based on what was declared by the World Health Organization that some herbal medicines do not have enough evidence of their potency and safety as people may think. In addition, they may be hazardous when combined with some modern medications.

Nearly half of the people in this study believe that using herbs and prescribed medicine is better than using one of them alone, while one of the studies showed that nationwide it is also preferred to use herbal medicine over the use of pharmaceutical drugs [17].

Another study showed that 86% of the population prefers prescribed medicine; similarly, this study shows that 69.9% of patients prefer prescribed medicine over herbal [18].

Moreover, 41.6% like to use herbs when they are sick without doctor seeking or prescribed medicine. The use of herbal remedies along with prescribed medications or without doctor seeking and knowing has been reported in some studies as standard practice by patients with chronic diseases and common diseases [19,20].

This study might not be the first to be done in the country, but it is presumed to be one of the few conducted in the western region. A multiregional study that was done in 2018 including 809 random samples of adults from most regions of the country showed that 88.4% use or have used herbal medicine before [21]. Comparatively, our study showed that an overall 93.7% of the random sample have used herbs before whether it was for themselves or their family. In the multiregional study, it was shown that 48.2% of participants took information about herbs from family and friends [21]. Similarly, our study also showed that 57.8% of participants acquired most of their information about herbs from family and friends [21].

Another similarity that both studies found out is that most herb users are females accounting for 85% in the multiregional study and 53.2% in our study [21].

This study was limited by being conducted in one hospital in Jeddah. The cross-sectional study design did not allow assessment of the impact of herbal use on disease outcomes. The study also represented patients who visited the outpatient clinics with no information on the experience of inpatients.

## Conclusions

The use of herbal medicine among the Saudi population is a common practice and is associated with the female gender, adult population, and low level of education. The usage of herbs is strongly associated with the family member's use. The most used herbs were anise, cumin, cinnamon, and these were used commonly to treat conditions and disorders in the respiratory, GI, and endocrine. Also, patients prefer to visit physicians rather than using herbs themselves. However, there are a group of people who use herbs without medical consultation. A study on more hospitals in all regions of Saudi Arabia would better assess the attitudes and knowledge of herbal usage. A longitudinal multicenter study representing different regions in Saudi Arabia is recommended to further assess sociocultural factors that contribute to the use of herbs and measure health outcomes in different age groups and medical conditions.
